# Pigs Like It Varied; Feeding Behavior and Pre- and Post-weaning Performance of Piglets Exposed to Dietary Diversity and Feed Hidden in Substrate During Lactation

**DOI:** 10.3389/fvets.2019.00408

**Published:** 2019-11-19

**Authors:** Anouschka Middelkoop, Manon A. van Marwijk, Bas Kemp, J. Elizabeth Bolhuis

**Affiliations:** Adaptation Physiology Group, Department of Animal Sciences, Wageningen University and Research, Wageningen, Netherlands

**Keywords:** behavior, creep feed, dietary diversity, enrichment, feed intake, foraging, piglet, weaning

## Abstract

Timely intake of solid feed is essential to ease the nutritional change from sow's milk to solid feed at weaning and thereby to reduce weaning-related problems. A significant percentage of piglets, however, do not or hardly consume solid feed before weaning. We studied effects of dietary variety and presenting the feed in substrate during lactation on the feeding behavior and performance of piglets up to 2 weeks post-weaning. Feed was provided *ad libitum* from d4 in two feeders, with four bowls each. In a 2 × 2 arrangement, 40 litters received either creep feed as a monotonous diet (MO) or four feed items simultaneously, i.e., creep feed, celery, cereal honey loops and peanuts, as a diverse diet (DD) and the feed was either provided without (CON) or with substrate (SUB), i.e., sand, in one of the two feeders up to weaning. Dietary diversity highly stimulated feed exploration and eating (≥2.5 times), feed intake and the percentage of (good) eaters from early in lactation, and enhanced piglet growth toward weaning (by 29 g/d), although MO-piglets spent more time eating creep feed from d18. Within MO, SUB-litters consisted of more good eaters than CON-litters. At weaning (d28) four piglets from the same treatment were grouped (*n* = 40 pens). DD-CON had the highest post-weaning feed intake and gain between d5–15 and the lowest proportion of pigs with higher tail damage scores. However, effects regarding behavior remained inconclusive, as DD-piglets had a lower and higher number of body lesions at 4 h and d15 post-weaning, respectively, spent less time exploring the feed(er) and drinker and environment, and more time nosing pen mates than MO-piglets. SUB-piglets showed a reduction in total post-weaning feed intake, gain (particularly between d0–2) and inactivity, increased levels of manipulation and aggression at week 1 and a higher number of body lesions at 4 h and d15 post-weaning. In conclusion, dietary diversity seems a promising feeding strategy in getting piglets to eat during lactation. Provision of substrate in the feeder subtly stimulated foraging behavior, but negatively impacted post-weaning adaptation, probably because treatments were not reinforced after weaning and piglets thus experienced loss of enrichment.

## Introduction

In most commercial pig farms, piglets are weaned at 3–4 weeks of age by separating them from the sow. This early and abrupt weaning is commonly seen as the most stressful event in a pig's life, as it comprises multiple concurrent stressors, such as changes in environment, social structure and diet. Several studies have shown that stimulating the natural behavior of pigs benefits their capacity to deal with weaning. Firstly, a stimulus-rich environment has been shown to enhance explorative behaviors and reduce weaning stress and weaning-stress-induced behaviors compared to a barren farrowing and weaner pen [more space and wood shavings, peat, branches, and straw on the floor: (1); tray of peat: (2); box with wood bark: (3)]. Secondly, loose housing of sows (1) and group housing of sows and their litters during lactation (4) were found to reduce damaging oral manipulation of pen mates and increase play behavior of piglets after weaning. Moreover, group housing of sows and their litters reduced behavioral signs of fear in piglets exposed to a novel environment (5). In addition, loose housing of sows plus the provision of sawdust on the floor increased average daily gain of piglets in the first 2 weeks post-weaning and reduced belly nosing in piglets weaned at 3 weeks of age (6). Loose and group housing of sows resemble the social situation in nature, with pigs living in families (7, 8), in which information regarding what, where and how to eat is transmitted from sow to piglets (9, 10). Thirdly, outdoor-reared piglets have been reported to consume almost twice as much creep feed from 2 weeks of age compared to indoor-reared piglets (11). Moreover, even when not provided with creep feed, outdoor-reared piglets spent more time eating solid feed before weaning, such as sow feed and vegetation (12, 13), spent more time eating creep feed, which was novel to them, in a novel environment on the day of weaning (14), and spent more eating solid feed in the first hours after weaning compared to indoor-reared piglets which received creep feed (12, 15). In addition, outdoor-reared piglets displayed less agonistic behavior, belly nosing and other oral-nasal interactions than indoor-reared piglets (12, 13). It can be concluded that outdoor-reared piglets are more experienced with solid feed before weaning compared to indoor-reared piglets, of which a significant proportion does not or hardly consume solid feed before weaning (16, 17). This low feed intake before weaning, or even no feed intake at all, often results in a low feed intake after weaning (18, 19), accompanied by gastro-intestinal problems [reviewed by (20)], gut microbiota dysbiosis [reviewed by (21)], reduced weight gain and an increase in damaging behavior (22, 23). Since rearing environments for commercial indoor production are different from (semi-)natural conditions, strategies that stimulate the natural feeding behavior of indoor-reared piglets may improve their weaning transition, as the intake of solid feed before weaning correlates with the intake of feed after weaning (24, 25).

In a (semi-)natural environment, pigs spend more than half of their active time on foraging and eating, of which mostly grazing, rooting and nosing [weaned and adult pigs: (26); from 8 weeks of age: (27)]. Their diet is diverse and consists of a variety of feed items such as grasses, fruits, nuts, fungi, leaves, insects, resin and roots [(26, 28), reviewed by (29)]. Already from a few days after birth, piglets have been observed foraging for other feed items than sow's milk, by digging soft soil and exploring and sampling leaves, mushrooms, acorns and corn (7, 8, 27) until they are exclusively feeding on solid feed between 8.5 and 22 weeks of age (30, 31). The foraging behavior of wild piglets peaks around 4 weeks of age and therefore precedes the development of ingestive behavior such as grazing, which mainly develops during week 4 of age (27). Foraging is the “appetitive phase” of feeding, which brings the pig into contact with feed. It concerns active, flexible, searching behaviors for food and indicates the need to satisfy appetite, but also has an important role in information gathering. Eating is the “consummatory phase” of feeding and is the achievement (“consummation”) of the goal and ends the appetitive foraging behavior (32, 33). For example, pigs need to shovel and root the ground, and therefore “work” for their feed, before roots and worms can be consumed (26).

In contrast with (semi-)natural conditions, indoor-reared pigs are generally offered a monotonous pre-mixed diet, that is freely available and often provided *ad libitum*. However, they still spend more than half of their active time on eating and redirected foraging toward the floor and pen, of which mostly rooting and nosing [weaned piglets of 4–6 weeks of age: (1)], suggesting indoor-reared pigs are highly motivated to forage, also in absence of hunger and/or nutrient deficiencies. In addition, some pigs are willing to work hard for access to rooting material (34) and behavioral demand studies have shown a low elasticity in demand for rooting material (35). These data suggest that the performance of foraging behavior on its own is rewarding to pigs, but foraging may be more rewarding when linked to feed intake. This is supported by preference tests in which pigs spent more time in an environment with feed hidden in substrate compared to an environment with feed and substrate offered separately (36) and spent more time with feed hidden in substrate compared to substrate only, although fed *ad libitum* (36, 37). Translating these findings to piglet rearing before weaning, supplementing solid feed with substrate may encourage suckling piglets to spend more time at the feeder to perform foraging behavior and to consume feed. We also suggest that presentation of a more diverse diet stimulates the feeding behavior of piglets, by increasing exploratory behavior toward the feed and by reducing “sensory-specific satiety,” which is the decline in liking of eaten feed in comparison to other non-eaten feed [as reviewed by (38)]. Indications have been found that dietary variety, i.e., feeds that differ in at least one sensory domain (39), can indeed increase feed intake in humans [e.g., (40)], rats [e.g., (41–43)], and sheep [e.g., (44)]. This is also shown in pigs by providing feeds differing in flavor successively (45, 46) or simultaneously (46).

In this study, we therefore aimed to investigate the effect of dietary diversity (vs. dietary monotony), the effect of presenting the feed in a foraging-stimulating context (in substrate or not) and their potentially interacting effects on the foraging and ingestive behavior of suckling piglets, as well as their adaptive capacity to deal with weaning. It is hypothesized that dietary diversity and feed presentation in a foraging-stimulating context would positively affect feed intake prior to weaning and thereby increase post-weaning feed intake and body weight gain and reduce diarrhea and weaning-stress-induced behaviors.

## Methods

The protocol of the experiment (AVD104002016515) was approved by the Animal Care and Use committee of Wageningen University and Research (Wageningen, Netherlands) and in accordance with the Dutch law on animal experimentation, which complies with the European Directive 2010/63/EU on the protection of animals used for scientific purposes. The use of Indigo carmine as colorant in the feed was approved by the Medicines Evaluation Board (Utrecht, Netherlands).

### Animals, Housing, and Management

The study was set up as a 2 × 2 factorial arrangement (see below for treatments). Forty multiparous pregnant sows (range parity: 2–8; inseminated by Tempo boar semen, Topigs Norsvin, Vught, Netherlands) were divided over two farrowing rooms (balanced for treatments) in three consecutive batches (*n* = 10 sows per treatment). In total, 24 Topigs-20 sows, and 16 Norwegian Landrace × Topigs-20 sows were used, equally assigned to treatment groups. Sows originated from one conventional farm and were housed at research facility Carus (Wageningen University and Research, Netherlands) from 2 weeks before farrowing onwards and were crated in farrowing pens from 1 week before farrowing onwards until weaning. Sows were fed commercially available diets for gestation and lactation (Coppens Diervoeding, Helmond, Netherlands) twice a day, at 7:30 and 16:00 h. Feed was provided in portions and feed remains were removed within 30 min after feeding to prevent piglets from eating sow feed.

The farrowing pen (2.85 × 1.80 m) was equipped with a crate (2.85 × 0.60 m) including a feed trough, drinking nipple, a jute sack around farrowing and three chew objects that alternated two times a week (metal chain with either one of three attachments: bolts, ball, or PVC pipe) for the sow. It also included a drinking nipple, chew object (metal chain with bolts) and heating lamp (until day 13 of lactation) for the piglets. The floor consisted of slats and a rubber mat (1.75 × 1.20 m) that served as nest for the piglets and provided lying comfort to the sow. Within 24 h after birth, piglets received an ear tag and intramuscular iron injection and their birth weight and sex were determined. No teeth clipping, tail docking or castration were performed. Litter size was standardized to 13–15 piglets per litter by cross-fostering within 3 days of age, resulting in an average number of 13.5 ± 0.1 piglets per litter at d4.

**Figure 1:**
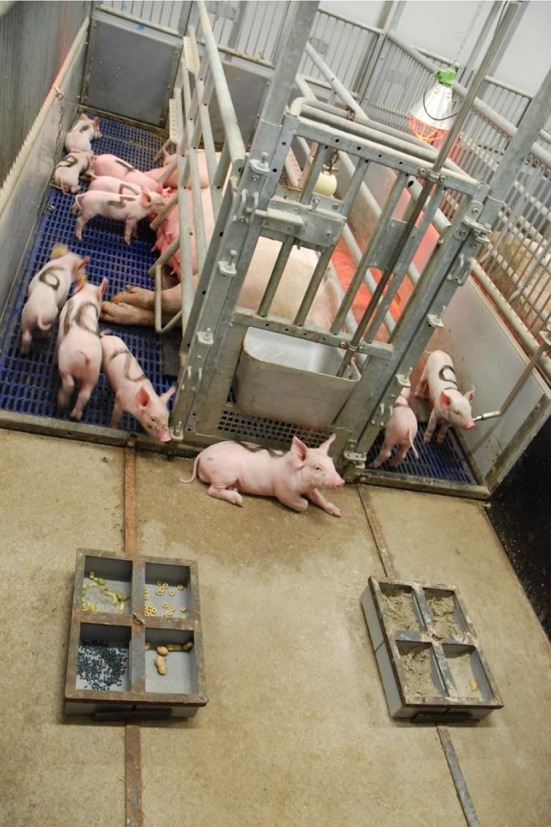
Set-up of the farrowing pen. From 4 days of age piglets had access to a concrete piglet feeding area, in front of the sow, including two feeders with four feeding spaces each.

The forty sows and their litters were allotted to one of four treatments at d4 based on sow breed and parity, and piglet birthdate and body weight at d0 and 4 after birth. From d4, piglets got access to a concrete piglet feeding area (1.37 × 1.80 m), in front of the sow, including two feeders with four bowls/feeding spaces each (17.5 × 13.5 cm per bowl) (Figure 1). In the farrowing rooms lights and radio were on from 7:00 to 19:00 h and lights were dimmed during the night. The room temperature was set to gradually decrease from 25°C around farrowing to 21°C at weaning. The parity of the sows (on average 5.2 ± 0.3) and litter size at weaning (on average 12.9 ± 0.2 piglets/litter) did not differ between treatments.

At weaning, i.e., 28.5 ± 0.2 days of age, a subset of 160 piglets (*n* = 10 weaner pens per treatment) was relocated in two weaner rooms (balanced for treatments) in two consecutive batches. Piglets were mixed with conspecifics from the same pre-weaning treatment and housed in pens with four piglets, of which two males and two females, which derived from three litters. Piglets were selected based on their sex and their body weight at d26 (close to the average weight of the litter and treatment group). Piglets with a history of leg/claw problems were excluded from selection. All weaner pens were identical and equipped with a feed trough (12 × 50 cm with three feeding places), drinking trough and chew object (metal chain with bolts). The flooring was slatted with a rubber mat (1.75 × 1.20 m) that provided lying comfort and prevented spillage of feed through the slats. Piglets were fed a commercially available nursery diet *ad libitum* (3-mm pellet, 161 g crude protein, 48 g crude fiber, and 11.8 g standardized ileal digestible lysine/kg dry matter, Coppens Diervoeding, Helmond, Netherlands). In the weaner rooms lights and radio were on from 7:00 to 19:00 h and room temperature was set to gradually decrease from 25°C at weaning to 23°C at 2 weeks post-weaning, when the experiment ended (d43). The experiment took place from May until August and the room temperature of the farrowing rooms in batch 3 and weaner rooms in batch 2 exceeded the settings as result of a heat wave from 15th of July until 7th of August 2018. A maximum temperature of 30 and 31°C were measured in these farrowing and weaner rooms, respectively.

### Feeding Strategies of Piglets During Lactation

**Figure 2:**
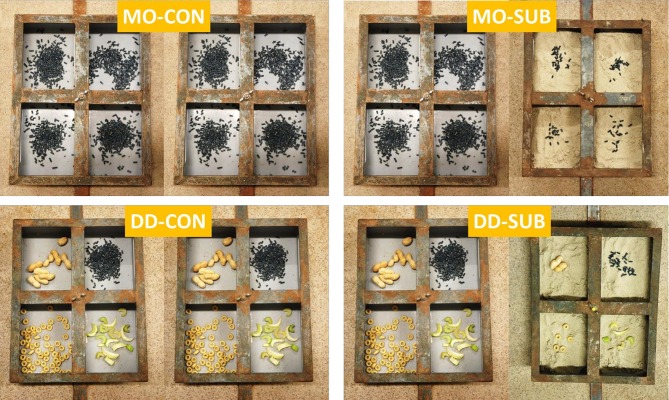
Litters were either provided with creep feed as a monotonous diet (MO) or four feed items simultaneously (creep feed, peanuts, celery, and cereal honey loops) as a diverse diet (DD). The feed was presented without (CON) or with substrate (SUB), which was sand, in one of two feeders. The feed was hidden in the substrate, but a few items were put on top of the substrate in the first feeding phase from d4 to d12 (pictures were taken in this phase).

Piglets were assigned to one of four treatment combinations in a 2 × 2 arrangement, with dietary variety (DV) and feed presentation (FP) during lactation as experimental factors. Piglets received either one solid feed item, i.e., creep feed, as a monotonous diet (**MO**) or received four solid feed items simultaneously (creep feed, celery, cereal honey loops, and peanuts in shell) as a diverse diet (**DD**) and the feed was either presented without substrate (**CON**) or with substrate (**SUB**) in one of the two feeders to stimulate natural foraging behavior (Figure 2). In the SUB-treatment, the feed in one of the feeders was hidden in the substrate, which was play sand, but a few items were put on top of the substrate from d4 to d12 only. Bowls were checked at least two times a day from d4 (7:30 and 16:00 h) and at least four times a day from d12 (7:30, 12:00, 16:00, and 17:30 h) to provide feed *ad libitum*. Bowls were refilled with feed using cups, in which the volume of the different feed items of DD were equal to each other. Feed in sand was provided in a ratio of one volume of feed in 12 volumes of sand. As feed intake increased with age, the ratio was decreased to one volume of feed in six volumes of sand from d19 onwards. The location of the feeder with sand, provided either left or right, was alternated over pens. The location of the feed items within the feeders, of which each item was provided in one of the four bowls per feeder, was alternated over pens and changed after each feed weigh-back (d12, 19, and 23) in a balanced order to capture all four possible positions during lactation. This was done by repositioning of the bowls. Each feed item was thus fed in the same bowl throughout lactation to prevent possible mixing of flavors.

The feed items were carefully chosen to create a diet as varied as possible in sensory properties (e.g., sweet and bitter taste, small and big sized items, crispy and sticky texture, hard and soft structure, smooth and ribbled surface) and to stimulate foraging such as extraction and chewing. Peanut shells (~3.8 × 1.3 cm) were cracked up to day 12 to ease opening up of the shell by the piglets. Celery leaves were removed and the celery was cut in pieces of ~2.5 × 2 × 0.6 cm. To maintain freshness of the feed, feed bowls were cleaned using paper towels and water and all feed items were replaced after each feed weigh-back. In addition, celery in the feed bowls was refreshed every other day. Cereal honey loops (1.5 × 0.5 cm) and creep feed (3 mm in diameter) presented in sand were refreshed daily. Peanuts, celery, cereal honey loops (Supplementary Table S1) and sand were purchased from local suppliers. Sand was chosen as substrate, as more edible substrates like wood shavings and peat provide some diversity to the diet and may affect the gut microbiota population of piglets. The creep feed (11.8 MJ/kg as-fed net energy, 195 g crude protein, 11.9 g standardized ileal digestible lysine/kg dry matter) was pelleted by Research Diet Services (Wijk bij Duurstede, Netherlands). The creep feed was high in dietary non-starch polysaccharides (261 g/kg dry matter), originating from cereal grains, sugarbeet pulp, oat hulls, galacto-oligosaccharides, inulin, and high-amylose starch and included 5 g/kg of feed colorant Indigo carmine (E132 Eurocert 311811, Sensient Food Colors, Elburg, Netherlands; Supplementary Tables S2, S3).

### Measurements

#### Piglet Behavior and Identification of Eaters (Based on Behavioral Observations)

Piglets were individually marked using dark permanent hair dye pre-weaning and animal marking spray post-weaning. Feed-related behaviors in the farrowing pens were observed live at d11, 18, and 27 using 2-min instantaneous scan sampling for six sessions of 1 h per day (i.e., 180 scans/piglet/d) using pen and paper. The ethogram with behaviors of interest in the farrowing rooms is given in Table 1. Observations in the farrowing rooms were used to calculate time spent on feed-related behaviors and to discriminate eaters from non-eaters at each observation day. Piglets were also classified into different eater classes according to Middelkoop et al. (47). Briefly, piglets that were observed eating on three observation days were classified as good eaters and piglets eating on two observation days as moderate eaters. Piglets eating on one observation day were classed as bad eaters and piglets that were never seen eating as non-eaters. If a piglet was scored as an eater based on behavioral observations, it was also investigated which feed items it consumed over the three observation days. Behaviors after weaning were recorded live on 35 and 42 days of age (d7 and 14 post-weaning) by 2-min instantaneous scan sampling for six 1-h sessions per day using a Psion hand-held computer with the Pocket Observer 3.1 software package (Noldus Information Technology, Wageningen, Netherlands). The ethogram with behaviors of interest in the weaner rooms is listed in Supplementary Table S4. The observation sessions in the farrowing rooms started at 8:00, 9:15, 10:30, 14:00, 15:30, and 16:45 h and in the weaner rooms at 8:00, 9:15, 10:30, 14:00, 15:15, and 16:30 h.

**Table 1:** Feeding behaviors during the suckling period of piglets that had access to two feeders with four feeding spaces/bowls each.

**Behavior**	**Description**
Exploring feeder	Sniffing, touching with snout, rooting, or chewing on the feeders
Exploring feed	Sniffing, touching with snout or rooting the content (feed or feed + sand) of the feeders (snout is in the feeder) or sniffing or touching feed on the floor (snout is outside the feeders)
Playing with feed	Rolling feed over the floor, walking around the pen with feed item in mouth or (energetically) shaking head while having feed item in mouth
Eating feed	Eating or chewing feed from the feeders or the floor
Suckling	Drinking milk from teat of sow (soft suckling noises)

*Litters were either provided with creep feed as a monotonous diet or four feed items simultaneously (one feed item per bowl) as a diverse diet. The feed was presented without or with substrate in one of two feeders. When given the choice between substrate or not, it was noted at/from which feeder (i.e., with sand, without sand, or unknown) the piglets were exploring/eating to study feed presentation preferences. When given the choice between different feed items, it was scored to which feed item (creep feed, celery, cereal honey loops, peanuts, or unknown) the behavior was directed to test feed item preferences*.

#### Identification of Creep Feed Eaters (Based on Rectal Swabs)

Rectal swabs were taken at d12, 19, 23, 26, and 28 to determine the intake of creep feed including feed colorant Indigo carmine qualitatively (yes or no) on piglet level at each measurement day. Piglets were also classified into different eater classes according to Collins et al. (17). In short, piglets of which blue color was present on the swab on four or five measurement days were classified as “good/early eaters.” “Moderate eaters” had blue color on the swab on two or three measurement days and “bad eaters” on only one measurement day. This may not necessarily concern the last measurement day(s) and therefore these piglets cannot be called “late eaters.” Swabs of “non-eaters” did not include blue color at any of the measurement days.

#### Feed Intake

Pre-weaning feed intake was determined in fresh weight between d4–12, d12–19, d19–23, and d23–28, thereby resulting in four feeding phases. It was measured on litter level per feeder (with or without substrate within SUB) and per feed item (within DD) by weighing feed remains from the feeders and floor (including peanut shells). Feed remains derived from SUB-pens were sieved before weighing them to minimize the attachment of sand, but sieving did not fully remove the sand attached to the feed items. To assess the effects of sand on the feed intake measures (attachment of sand to the items and moisture from the sand taken up by the items) we weighed the feed remains in two pens with no animals in it. In these “test pens” the feed was presented and handled as DD-SUB. In addition to pre-weaning feed intake, the number of refills (i.e., number of times extra feed was added to the bowls in the pen) were recorded on litter level per feeder (with or without substrate within SUB) and per feed item (within DD) as an estimate of the extra feed provided per feeding phase. The volume of the different feed items within DD were equal per refill and was 1:12 (feed:sand) up to d19 and 1:6 thereafter. Standard refreshment procedures (as mentioned for creep feed, celery, and cereal honey loops in sand) were not included in the number of refills. Post-weaning feed intake was determined on pen level between 0–4, 4–24, d1–2, d2–5, and d5–15 post-weaning. Feed wastage was kept to a minimum by placing the feeders on the solid floor in the farrowing and weaner pens.

#### Sow and Piglet Body Weight Development

Sow body weight and back fat thickness at left and right P2 positions were measured using an ultrasonic Renco Lean Meater (MS Schippers, Bladel, Netherlands) at 1 week before farrowing and at weaning. Piglets were individually weighed at d0 (within 24 h after birth), d4 (before commencing feeding), 19, 26, 28 (at weaning) before weaning and d1, 2, 5, and 15 after weaning (at the end of the experiment).

#### Body Lesions and Damage on Piglets

The number of body lesions on the piglets were monitored as a measure of aggression according to Turner et al. (48) at 4 h and d1, d2, and d15 post-weaning. Bite injuries on ears and tails of the piglets were classified into no damage, bite marks, small wound or medium wound as a measure of oral manipulation according to van Nieuwamerongen et al. (4) at d15 post-weaning.

#### Fecal Consistency Scores of Piglets

Feces in the weaner pens were scored daily for consistency according to Pedersen and Toft (49). Score 1 (firm and shaped) and 2 (soft and shaped) represent normal feces. Score 3 (loose) and 4 (watery) represent diarrhea. The highest fecal consistency score that was observed in a pen was selected on each measurement day and averaged over 2 weeks post-weaning to calculate the mean fecal consistency score per pen. Feces were removed on a daily basis after scoring to guarantee consistency scoring of fresh feces.

### Statistical Analyses

#### Data Processing

Results regarding pre-weaning feed intake from the “test pens” indicated that in the feeders with sand, feed intake calculations based on weighing back the feed may have overestimated the intake of celery and underestimated the intake of creep feed and cereal honey loops. In the end, we did not use these results to correct the feed intake measurements, as the “test pens” did not resemble closely enough the situation in pens with animals. Firstly the feed, and thus also celery, disappeared at a faster rate in pens with animals due to consumption. Secondly, sand was regularly rooted by the piglets and freshly added as required, and thirdly feed could get in contact with saliva due to chewing efforts of the piglets. Feed intake data of SUB-pens were therefore excluded from analyses.

Piglet behaviors in the home pen were averaged per piglet per day and expressed as proportions of time. Behavioral element “chewing feces” was excluded from analyses because it was seen very rarely (0.01% of observation time). The ear with the highest damage score (either the left or right ear of the piglet) was used in the analyses of ear damage. Only five piglets had small wounds on their ears and only one piglet had a medium ear wound, therefore ear damage was analyzed as 0–1 variable, i.e., no damage (0) vs. damage (1: bite marks + small wound + medium wound). Only four piglets had medium tail wounds, therefore small and medium tail wounds were combined into one score prior to data analyses. Fecal consistency score 1 and 2 were combined into one score prior to data analyses, as they both represent normal feces. The presence of outliers was tested by a Grubb's test and two outliers were excluded from further analyses, i.e., one piglet was excluded from the calculation of average daily gain between d19–26 and one piglet from the calculation of average daily gain between d26–28.

#### Data Analyses

Data were analyzed with generalized linear (GLIMMIX) and linear (MIXED) mixed models in the statistical software SAS 9.4 (SAS Institute Inc., Cary, NC, USA). Proportions of time spent on the different behaviors and the proportion of eaters per litter (based on home pen observations and blue colored rectal swabs) were analyzed in a GLIMMIX with a binomial distribution, logit link function and an additional multiplicative overdispersion parameter. The proportion of piglets playing with feed, the proportion of weaner piglets with tail damage and individual creep feed classification of piglets based on behavioral observations were analyzed in a Fisher's exact test, because there were empty classification categories scoring 0 only (i.e., 0 MO-piglets were playing with feed, 0 DD-CON piglets had bite marks on their tails and 0 DD-litters had non-eaters). Individual creep feed classification of piglets based on blue colored rectal swabs was analyzed in a GLIMMIX with a multinomial distribution and a cumulative logit link function. Subsequently, data on individual creep feed classification were expressed as binary data (good + moderate eaters vs. bad eaters + non-eaters) and analyzed in a GLIMMIX with a binary distribution and a logit link. The number of feed refills, the number of body lesions and the number days with post-weaning diarrhea were analyzed in a GLIMMIX with a Poisson distribution, log link function, and an additional multiplicative overdispersion parameter. The occurrence of watery diarrhea and ear damage were expressed as binary data and analyzed in a GLIMMIX with a logit link and binary distribution. Feed intake before weaning, average daily feed intake (ADFI), average daily gain (ADG), body weight (BW), uniformity in BW expressed as coefficient of variation, feed conversion ratio, mean fecal consistency score as well as sow BW and back fat loss were analyzed in a MIXED procedure. For feed intake and ADG, totals over the pre- (d4–28) and post-weaning period (d0–15 post-weaning) were analyzed, as well as effects on separate periods. Model residuals of the MIXED procedure were checked for normality. Creep feed intake between d4–12, creep feed intake between d4–28 and feed intake in the first 4 h after weaning were log transformed before analyses.

The models included the fixed effects of dietary diversity (DD vs. MO), feed presentation (SUB vs. CON), their interactions, as well as batch (batch 1, 2, or 3 before weaning and batch 1 or 2 after weaning). Treatment DD-SUB had 100% eaters at d27 based on home pen observations, therefore the interaction between DV × FP was excluded from the model at d27. In addition, for behavior, BW, ADG, and the number of body lesions, the model included a random pen effect, nested within treatments and batch (farrowing pen for observations pre-weaning and weaner pen for observations post-weaning). Back fat thickness at 1 week before farrowing was used as covariate in the analyses of back fat loss during lactation and CV at d4 was used as covariate in the analyses of CV at d28. Moreover, BW at weaning was used as covariate in the analyses of BW and ADG after weaning, but excluded from analyses when not significant.

To study the effects of sand (with or without sand in the feeder) within FP, fixed effects of sand, DV, and their interaction, as well as batch, were used. Furthermore, to test feed item preferences within DD, fixed effects of feed item and batch were used. Pen [nested within dietary treatment (DV or FP, respectively) and batch] was also used as random effect in the analyses within treatments.

Significant fixed effects were further analyzed using *post-hoc* pairwise comparisons of least squares means. Data are presented as (untransformed) means ± SEM based on pen averages. Differences at *P* < 0.05 were considered statistically significant and differences at 0.05 ≤ *P* < 0.10 were considered a trend.

## Results

### Feed-Related Behavior Before Weaning

**Figure 3:**
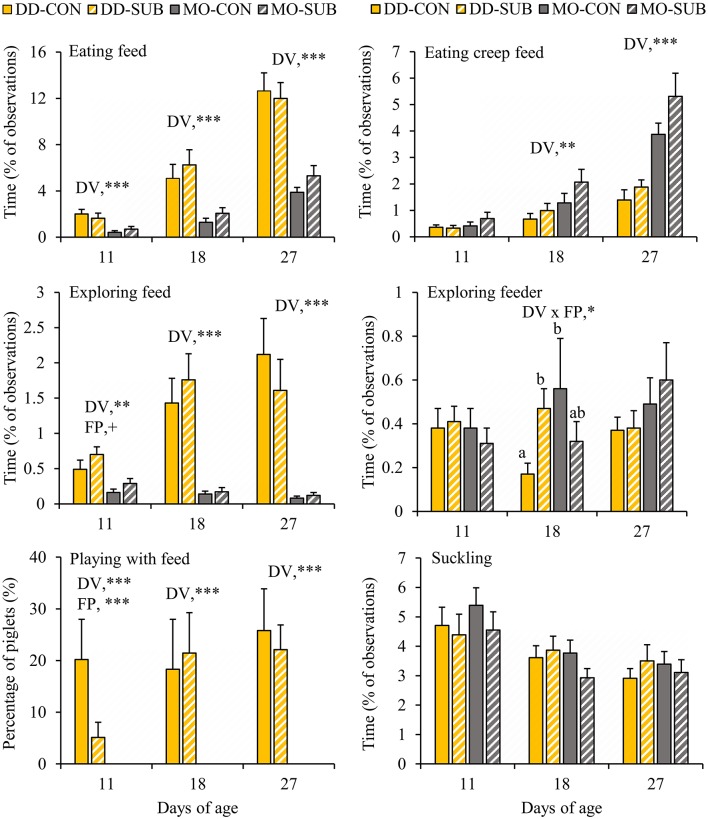
Feed-related behavioral activities (% of total observations) of litters provided with creep feed as a monotonous diet (MO) or four feed items simultaneously as a diverse diet (DD). The feed was presented without (CON) or with substrate (SUB) in one of two feeders. DV, dietary variety; FP, feed presentation. Data are expressed as means ± SEM based on pen averages. +, *, **, ***Significant effects at *P* < 0.10, < 0.05, < 0.01, < 0.001, respectively. Within a day superscripts without a common letter differ at *P* < 0.05.

DD-piglets spent at least two and a half times more time on feed exploration (DD vs. MO, d11: 0.60 ± 0.09 vs. 0.22 ± 0.05%; d18: 1.60 ± 0.25 vs. 0.15 ± 0.03%; d27: 1.87 ± 0.33 vs. 0.10 ± 0.02%) and eating (DD vs. MO, d11: 1.83 ± 0.29 vs. 0.55 ± 0.14%; d18: 5.66 ± 0.88 vs. 1.67 ± 0.31%; d27: 12.32 ± 1.02 vs. 4.59 ± 0.51%) than MO-piglets at all observation days in lactation (Figure 3). When looking at creep feed only, however, MO-piglets were seen eating this feed item two times more than DD-piglets at d18 (MO: 1.67 ± 0.31 vs. DD: 0.83 ± 0.17%) and d27 (MO: 4.59 ± 0.51 vs. DD: 1.63 ± 0.24%). Playing with feed was only shown by DD-piglets and not performed by MO-piglets (DD: 0.15 ± 0.03 vs. MO: 0% of the time). The percentage of piglets playing with feed was therefore 0% at all observation days in MO and differed from DD that had 12.6 ± 4.4, 19.9 ± 6.1, and 24.0 ± 4.6% of the piglets playing with feed at d11, 18, and 27, respectively. SUB-piglets tended to spend more time on feed exploration than CON-piglets at d11 (SUB: 0.50 ± 0.08 vs. CON: 0.32 ± 0.08% of time), but a lower proportion of SUB- (2.6 ± 1.5%) than CON-piglets (10.1 ± 4.5%) were seen playing with feed at this age. Interactions between DV x FP were found on exploration of the feeder at d18, indicating that DD-SUB piglets spent more time exploring the feeder than DD-CON piglets, whereas in MO piglets no significant differences were found between SUB and CON. Suckling was not affected by the treatments. Time spent exploring the feed positively correlated with time spent eating at all observation days (Piglet level: *r* = 0.42, 0.62, 0.65 at d11, 18, and 27, respectively; Litter level: *r* = 0.74, 0.76, 0.77; *P* < 0.0001 for all correlations).

### Percentage of Eaters Identified by Behavioral Observations

**Figure 4:**
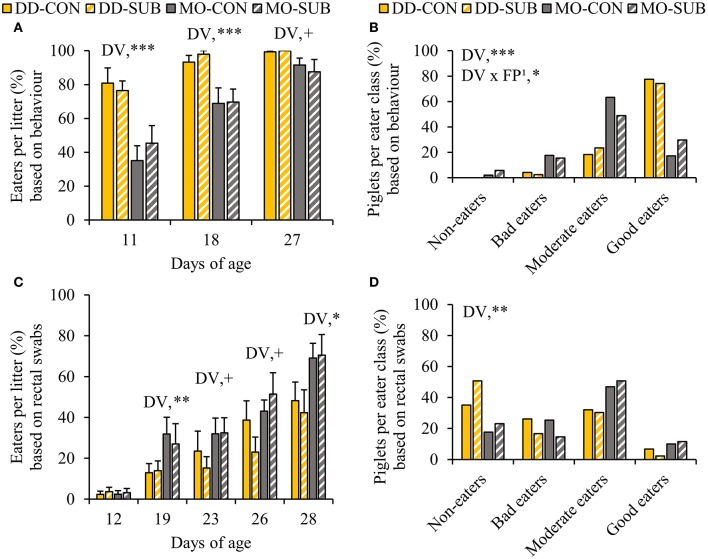
The percentage of eaters per litter over time and individual eater classification of piglets based on live home pen observations **(A,B)** and blue colored rectal swabs **(C,D)**. Litters were either provided with creep feed as a monotonous diet (MO) or four feed items simultaneously as a diverse diet (DD). The feed was presented without (CON) or with substrate (SUB) in one of two feeders. DV, dietary variety; FP, feed presentation. Data are expressed as means ± SEM based on pen averages. ^1^SUB effect within MO. +, *, **, ***Significant effects at *P* < 0.10, < 0.05, < 0.01, < 0.001, respectively.

DD enhanced the percentage of piglets within the litter observed to be eating at d11 and d18 and tended to at d27 compared to MO (DD vs. MO, d11: 78.7 ± 5.1 vs. 40.3 ± 6.7%; d18: 95.6 ± 2.3 vs. 69.3 ± 5.8%; d27: 99.6 ± 0.4 vs. 89.5 ± 4.1%, Figure 4A). In line with this, when we classified the piglets in each eater category, we found that DD resulted in a higher number of piglets in better eater classes (Figure 4B), as it enhanced the number of good eaters by three times compared to MO (DD: 75.8 vs. MO: 23.5%, *P* < 0.0001). Notably, all piglets within DD were observed to be eating on at least one observation day (DD: 100 vs. MO: 96% of the piglets). SUB also increased the number of piglets in better eater classes, but only within MO (SUB effect within MO: *P* = 0.02), showing more good eaters (MO-SUB: 29.8 vs. MO-CON: 17.2%, *P* = 0.02), and less moderate eaters (MO-SUB: 48.9 vs. MO-CON: 63.2%, *P* = 0.02).

### Percentage of Creep Feed Eaters Identified by Blue Colored Rectal Swabs

In accordance with behavioral observations of eating the creep feed (Figure 3), which included feed colorant Indigo carmine, the percentage of creep feed eaters per litter identified by blue colored rectal swabs was higher or tended to be higher in MO-litters compared to DD-litters from d19 onwards (MO vs. DD, d19: 29.5 ± 6.3 vs. 13.4 ± 3.2%; d23: 32.2 ± 5.2 vs. 19.3 ± 5.6%; d26: 47.3 ± 5.8 vs. 30.8 ± 6.1%; d28: 69.8 ± 6.1 vs. 45.3 ± 7.1%; Figure 4C). In addition, MO-piglets were more likely to be better creep feed eaters compared to DD-piglets (*P* < 0.01, OR = 4.27, 95% CI = 1.56–11.67, Figure 4D), particularly to be moderate and good creep feed eaters (moderate + good creep feed eaters, MO: 59.6 vs. DD: 35.7%, *P* < 0.01).

### Feed Intake Before Weaning

Feed in DD-pens (sum of all feed items) needed to be refilled more often compared to feed in MO-pens (12.7 ± 1.1 vs. 3.9 ± 0.4 refills/pen/d between d4–28) in all four feeding phases (double as much in phase 1, three times as much in phase 2 and four times as much in phase 3 and 4; Table 2). When looking at creep feed only, however, this feed item was refilled half the number of times in DD-pens than in MO-pens (2.0 ± 0.2 vs. 3.9 ± 0.4 refills/pen/d between d4–28). Based on weighing fresh weight of the feed remains, DD-piglets consumed 1,267 ± 169 g of feed during lactation (d4–28), of which 178 ± 34 g creep feed (2,839 kJ ME), while MO-piglets consumed 260 ± 38 g creep feed (4,147 kJ ME) during lactation. Besides, DD-piglets consumed 566 ± 84 g celery (340 kJ ME), 252 ± 47 g cereal honey loops (4,024 kJ ME) and 270 ± 54 g peanuts (7,058 kJ ME). DD-piglets thereby consumed 1 kg more in total during lactation than MO-piglets, but MO-piglets tended to consume more creep feed than DD-piglets before weaning. Although SUB-pens did not differ from CON-pens in the total number of refills, SUB-pens tended to be refilled more often with creep feed than CON-pens between d12–19 (2.8 ± 0.5 vs. 2.0 ± 0.3 refills/pen/d) and MO-SUB was more often refilled with creep feed than the other three treatments between d19–23.

**Table 2:** Feed intake during the suckling period, based on the number of refills and weighing feed remains.

	**DD**		**MO**		**Significance**		
	**CON**	**SUB**	**CON**	**SUB**	**DV**	**FP**	**DV × FP**
**Total number of refills (sum of all feed items), per pen/d**							
d4–12	3.7 ± 0.9	2.8 ± 0.7	1.8 ± 0.7	1.6 ± 0.5	**0.002**	0.50	0.72
d12–19	9.9 ± 1.5^a^	9.4 ± 1.6^a^	2.7 ± 0.4^b^	4.0 ± 0.9^b^	**<0.0001**	0.20	**<0.05**
d19–23	15.7 ± 2.8^a^	12.6 ± 1.9^a^	2.5 ± 0.4^b^	4.2 ± 0.8^b^	**<0.0001**	0.54	**0.09**
d23–28	35.1 ± 4.4^a^	27.0 ± 3.4^a^	7.1 ± 1.1^b^	10.0 ± 1.5^b^	**<0.0001**	0.86	**0.08**
Total, d4–28	14.1 ± 1.8^a^	11.4 ± 1.3^a^	3.3 ± 0.5^b^	4.5 ± 0.6^b^	**<0.0001**	0.72	**0.07**
**Number of creep feed refills, per pen/d**							
d4–12	0.8 ± 0.2	0.6 ± 0.2	1.8 ± 0.7	1.6 ± 0.5	**0.002**	0.77	0.96
d12–19	1.4 ± 0.3	1.7 ± 0.2	2.7 ± 0.4	4.0 ± 0.9	**<0.001**	**0.09**	0.46
d19–23	2.2 ± 0.4^a^	1.8 ± 0.3^a^	2.5 ± 0.4^a^	4.2 ± 0.8^b^	**0.005**	0.42	**0.02**
d23–28	5.0 ± 1.1	4.4 ± 0.8	7.1 ± 1.1	10.0 ± 1.5	**0.002**	0.62	0.21
Total, d4–28	2.1 ± 0.3	1.9 ± 0.2	3.3 ± 0.5	4.5 ± 0.6	**0.0001**	0.41	0.18
**Feed intake, g/piglet**							
d4–12	72 ± 17	–	9 ± 6	–	**<0.0001**	–	–
d12–19	206 ± 38	–	64 ± 17	–	**<0.001**	–	–
d19–23	291 ± 53	–	58 ± 14	–	**<0.001**	–	–
d23–28	696 ± 96	–	129 ± 18	–	**<0.001**	–	–
Total, d4–28	1267 ± 169		260 ± 38	–	**<0.0001**	–	–
**Creep feed intake, g/piglet**							
d4–12	6 ± 2	–	9 ± 6	–	0.82	–	–
d12–19	37 ± 10	–	64 ± 17	–	**0.07**	–	–
d19–23	44 ± 12	–	58 ± 14	–	0.49	–	–
d23–28	92 ± 21	–	129 ± 18	–	0.17	–	–
Total, d4–28	178 ± 34	–	260 ± 38	–	**0.08**	–	–

*Piglets were provided with creep feed as monotonous diet (MO) or four solid feed items (creep feed, celery, cereal honey loops, and peanuts) simultaneously as diverse diet (DD) before weaning and their pre-weaning diet was presented without (CON) or with substrate (SUB) in one of two feeders. DV, dietary variety; FP, feed presentation. Data are means ± SEM. Within a row superscripts without a common letter differ at P < 0.05. Significant P-values and trends are presented in bold*.

### Effects of Sand (With or Without Sand in the Feeder) Within Feed Presentation Strategy (SUB)

**Figure 5:**
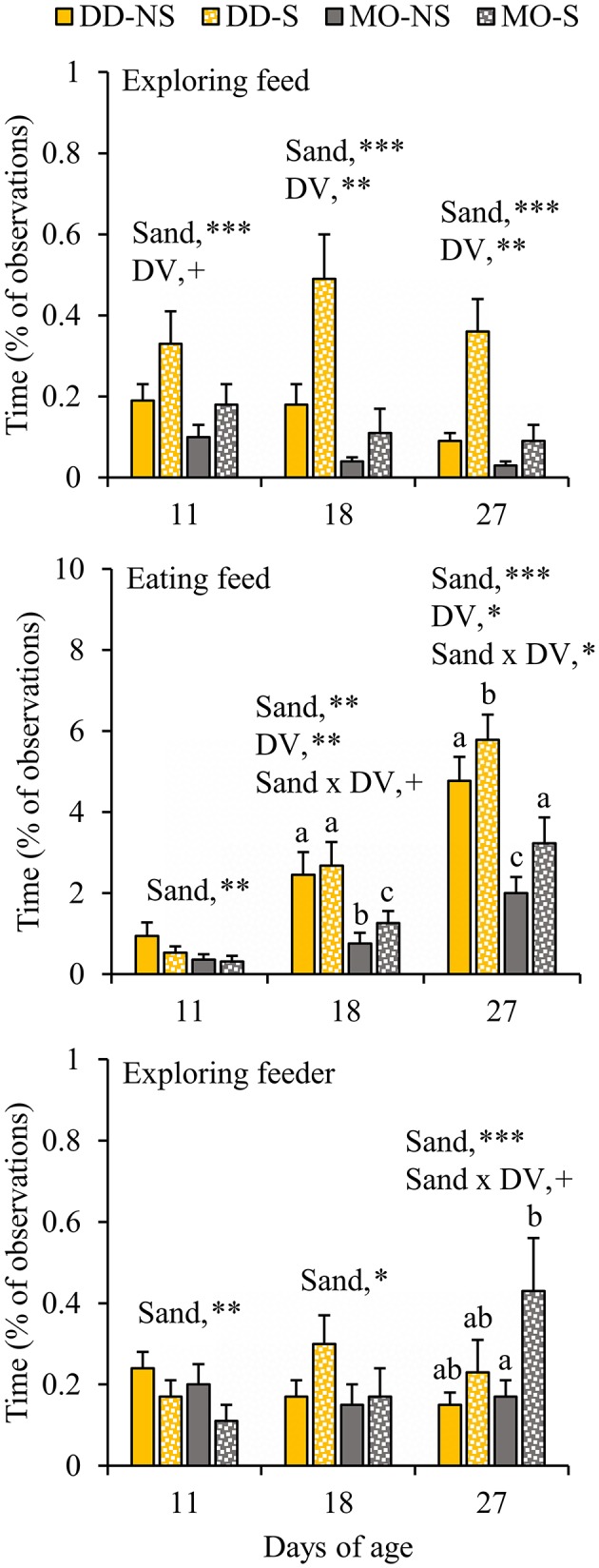
Feed-related behavioral activities (% of total observations) of litters that had their feed presented in one feeder without substrate (NS) and in a second feeder with substrate (S). The litters were either provided with creep feed as a monotonous diet (MO) or four feed items simultaneously as a diverse diet (DD). F, feeder; DV, dietary variety. Data are expressed as means ± SEM based on pen averages. +, *, **, ***Significant effects at *P* < 0.10, <0.05, <0.01, <0.001, respectively. Within a day superscripts without a common letter differ at *P* < 0.05.

When given the choice between sand in the feeder or not, piglets spent more time exploring the feed (plus sand) in the feeder with sand (S) compared to exploring the feed in the feeder without sand (NS) at all observation days (S vs. NS, d11: 0.25 ± 0.05 vs. 0.14 ± 0.03; d18: 0.30 ± 0.08 vs. 0.11 ± 0.03; d27: 0.22 ± 0.05 vs. 0.06 ± 0.02%; Figure 5). At d18, sand x DV tended to interact on eating feed, showing that SUB-piglets spent more time eating from the feeder with sand than from the feeder without sand when fed a monotonous diet. The interaction was significant at d27, and showed that the effect of sand was more pronounced within MO (*P* < 0.0001) than within DD (*P* = 0.02). Moreover, SUB-piglets spent more time exploring the feeder with sand than the feeder without sand at d18, and also at d27 when fed a monotonous diet. Vice versa was observed at d11, when SUB-piglets spent less time exploring the feeder with sand and eating from the feeder with sand than the feeder without sand (S vs. NS, exploring feeder at d11: 0.14 ± 0.03 vs. 0.22 ± 0.03%; eating feed at d11: 0.42 ± 0.10 vs. 0.65 ± 0.19%). In agreement, between d4–12, the feeder with sand was refilled less often compared to the feeder without sand (0.8 ± 0.2 vs. 1.4 ± 0.3 refills/pen/d, *P* = 0.003), but no differences in the number of refills were observed between the feeder with and without sand from d12 onwards (data not shown) and in total between d4–28 (S vs. NS: 3.9 ± 0.5 vs. 4.1 ± 0.6 refills/pen/d, *P* = 0.95).

### Effects of Feed Item Within Dietary Diversity (DD)

**Figure 6:**
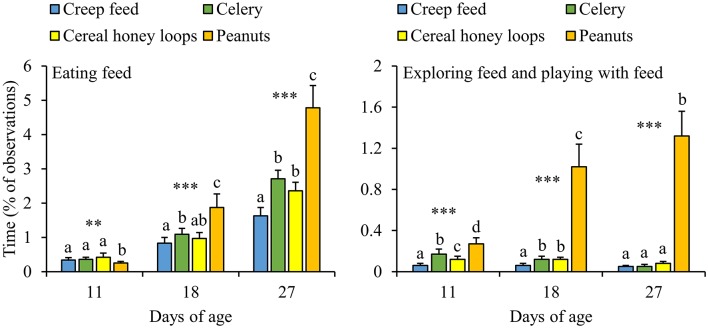
Feed-related behavioral activities (% of total observations) toward four different feed items of litters provided with those four feed items simultaneously as a diverse diet. Data are expressed as means ± SEM based on pen averages. **, ***Significant effects at *P* < 0.01 and <0.001, respectively. Within a day superscripts without a common letter differ at *P* < 0.05.

The majority of DD-piglets, i.e., 86%, was seen eating all four feed items before weaning, whereas 10% was seen eating three feed items, 3% two feed items and 1% only one out of four feed items. Some piglets developed strong feed preferences for either one feed item (e.g., piglet G43: 0.6, 10.0, 1.7, and 1.1% of the observation time eating creep feed, celery, cereal honey loops and peanuts, respectively, at d27) or multiple (e.g., piglet F43: 4.4, 10.6, 1,1, 13.3%, respectively), while others divided their feeding time equally over the feed items (e.g., piglet B18: 2.8, 2.2, 2.2 and 3.3%, respectively). Strong feed preferences were also observed between pens, with pens that mainly consumed one feed item (e.g., pen 3.14.2: 18, 25, 115, and 36 refills of creep feed, celery, cereal honey loops, and peanuts, respectively between d23 and 28), two or three feed items (e.g., pen 1.15.1: 34, 62, 64, 32 refills, respectively) vs. pens that divided their feeding time equally (e.g., pen 1.15.7: 47, 57, 51, and 48 refills, respectively). Overall, DD-piglets spent more time eating peanuts and exploring plus playing with peanuts than with the other three feed items (Figure 6). Only at d11, DD-piglets spent less time eating peanuts compared to the other three feed items. Next to peanuts, piglets spent less time eating creep feed than celery and spent less time exploring creep feed than celery and cereal honey loops at d18. Piglets spent as well less time eating creep feed than celery and cereal honey loops at d27. In addition, all feed items differed in the amount of time that was spent on exploring plus playing toward them at d11, with the lowest amount of time exploring plus playing toward creep feed, followed by cereal honey loops, celery, and the largest amount of time toward peanuts. Comparing the different feed items in DD-pens in terms of the number of refills, no differences were found in how often the different feed items were refilled in the first feeding phase (data not shown, *P* = 0.22). From the second feeding phase onwards, pellets were refilled half the number of times than peanuts, celery and cereal honey loops in DD-pens (data not shown, *P* < 0.01 for all phases). In total before weaning, pellets were refilled less often than the other three items (2.0 ± 0.2, 3.8 ± 0.3, 3.7 ± 0.5 and 3.3 ± 0.4 refills/pen/d of creep feed, celery, cereal honey loops, and peanuts, respectively, between d4 and 28, *P* ≤ 0.001).

### Sow and Piglet Body Weight Development

The number of weaned piglets and sow body weight and back fat loss during lactation were not affected by dietary variety, feed presentation, or their interaction (Table 3). Dietary variety and feed presentation tended to interact on ADG between d4 and 19 (*P* = 0.06), in which DD-SUB piglets grew faster than MO-SUB piglets (*P* = 0.01). DD-piglets grew faster in the last 2 days prior to weaning compared to MO-piglets (DD: 280 ± 10 vs. MO: 251 ± 11 g/d), but the treatment groups did not differ in weaning weight at d28 and homogeneity in weaning weight within litters (CV). Time spent eating at d27 positively correlated with ADG between d26 and 28 in MO-piglets (*r* = 0.40; *P* < 0.0001 on piglet level and *r* = 0.74; *P* = 0.0002 on litter level), but not in DD-piglets (*r* = 0.05; *P* = 0.44 on piglet level and *r* = 0.31; *P* = 0.18 on litter level).

**Table 3:** Performance of sows and piglets during lactation.

	**DD**		**MO**		**Significance**		
	**CON**	**SUB**	**CON**	**SUB**	**DV**	**FP**	**DV × FP**
Sow back fat loss, mm	4.3 ± 0.5	6.3 ± 0.9	5.1 ± 0.8	4.4 ± 0.7	0.59	0.25	0.57
Sow body weight loss, kg	35 ± 3	39 ± 3	43 ± 5	37 ± 3	0.42	0.85	0.29
**ADG, g/piglet/d**							
d 4–19	210 ± 8^ab^	225 ± 6^a^	209 ± 8^ab^	198 ± 11^b^	**0.06**	0.86	**0.06**
d 19–26	223 ± 14	210 ± 14	205 ± 10	217 ± 11	0.66	0.96	0.39
d 26–28	292 ± 13	269 ± 15	244 ± 15	257 ± 16	**0.04**	0.77	0.33
Total, d 4–28	220 ± 8	224 ± 8	211 ± 9	208 ± 9	0.13	0.99	0.53
**Body weight, kg**							
d0	1.38 ± 0.05	1.38 ± 0.07	1.40 ± 0.07	1.44 ± 0.09	0.53	0.84	0.79
d4	1.86 ± 0.09	1.85 ± 0.10	1.86 ± 0.09	1.86 ± 0.10	0.93	0.99	0.89
d28	7.17 ± 0.25	7.22 ± 0.24	6.95 ± 0.25	6.86 ± 0.27	0.21	0.96	0.65
**Litter CV in BW, %**							
d4	20.0 ± 1.5	19.1 ± 0.8	18.2 ± 1.4	16.7 ± 1.4	**< 0.10**	0.41	0.88
d28	19.0 ± 1.3	18.3 ± 1.4	18.1 ± 1.2	19.2 ± 2.5	0.48	0.55	0.32

*Litters were either provided with creep feed as a monotonous diet (MO) or four feed items simultaneously as a diverse diet (DD). The feed was presented without (CON) or with substrate (SUB) in one of two feeders. DV, dietary variety; FP, feed presentation. Data are means ± SEM based on pen averages. Within a row superscripts without a common letter differ at P < 0.05. Significant P-values and trends are presented in bold*.

### Piglet Behavior After Weaning

#### Ingestive Behavior

Time spent eating tended to be higher in SUB-piglets compared to CON-piglets at week 1 post-weaning (SUB: 12.6 ± 0.8 vs. CON: 11.3 ± 0.6%; Table 4). The treatments tended to interact in their effect on time spent drinking at week 2 post-weaning (Table 5), but no significant differences were observed using *post-hoc* pairwise comparisons of least squares means.

**Table 4:** Behavioral activities (% of total observations) at week 1 after weaning (35 days of age).

**Behavior at week 1 after weaning**	**DD**		**MO**		**Significance**		
	**CON**	**SUB**	**CON**	**SUB**	**DV**	**FP**	**DV x FP**
**“Ingestive behavior”**							
Eating feed	11.7 ± 1.0	11.9 ± 0.7	10.8 ± 0.7	13.4 ± 1.4	0.91	**0.06**	0.11
Drinking	1.0 ± 0.2	0.7 ± 0.1	1.1 ± 0.2	1.0 ± 0.1	0.23	0.16	0.47
**“Exploratory behavior”**							
Exploring feed(er) and drinker	2.4 ± 0.3	2.4 ± 0.2	2.6 ± 0.3	3.8 ± 0.4	**0.03**	0.11	0.13
Exploring environment	19.4 ± 1.6	20.2 ± 1.2	24.9 ± 1.5	24.5 ± 2.2	**< 0.01**	0.84	0.76
Nosing environment	9.8 ± 0.7	10.8 ± 0.7	9.8 ± 1.0	10.0 ± 0.8	0.43	0.39	0.68
Rooting environment	1.2 ± 0.2	1.6 ± 0.4	1.8 ± 0.4	1.6 ± 0.3	0.23	0.79	0.21
Chewing environment	6.9 ± 1.2	5.8 ± 0.9	10.8 ± 1.2	10.2 ± 2.2	**<0.01**	0.41	0.85
Chewing air	1.6 ± 0.3	1.9 ± 0.3	2.5 ± 0.3	2.7 ± 0.3	**0.01**	0.39	0.60
**“Postures and locomotion”**							
Inactive behavior	48.5 ± 2.9	44.9 ± 2.7	44.0 ± 2.6	39.8 ± 3.1	**0.05**	**0.04**	0.63
Standing and walking	5.5 ± 0.7	7.5 ± 0.8	6.5 ± 1.0	6.7 ± 1.0	0.98	0.14	0.33
**“Pig-directed behavior”**							
Nosing pen mates	4.1 ± 0.4	4.4 ± 0.4	3.2 ± 0.3	3.0 ± 0.4	**<0.001**	0.76	0.62
Ear biting	0.5 ± 0.1	0.5 ± 0.1	0.4 ± 0.1	0.5 ± 0.2	0.63	0.59	0.55
Tail biting	0.4 ± 0.1	0.5 ± 0.1	0.4 ± 0.1	0.6 ± 0.1	0.98	0.17	0.59
Belly nosing	0.2 ± 0.1	0.1 ± 0.1	0.3 ± 0.2	0.2 ± 0.1	0.61	0.43	0.61
Manipulating pen mates	0.4 ± 0.1	1.0 ± 0.2	0.6 ± 0.1	0.7 ± 0.1	0.87	**0.02**	0.28
Mounting pen mates	0.8 ± 0.1	0.9 ± 0.4	0.6 ± 0.2	0.8 ± 0.2	0.50	0.28	0.60
Aggression	0.1 ± 0.03	0.2 ± 0.04	0.1 ± 0.1	0.2 ± 0.1	0.83	**0.03**	0.94
**“Other behavior”**							
Playing	3.4 ± 0.5	3.0 ± 0.3	2.8 ± 0.4	3.6 ± 0.5	0.99	0.66	0.24
Comfort behavior	0.6 ± 0.1^ab^	0.7 ± 0.1^a^	0.7 ± 0.1^a^	0.3 ± 0.1^b^	0.10	0.24	**0.02**
Eliminating	0.9 ± 0.1	0.9 ± 0.1	1.1 ± 0.1	0.8 ± 0.1	0.95	0.21	0.29

*Piglets were provided with one solid feed item as monotonous diet (MO) or four solid feed items as diverse diet (DD) before weaning and their pre-weaning diet was presented without (CON) or with substrate (SUB) in one of two feeders. DV, dietary variety; FP, feed presentation. Data are means ± SEM based on pen averages. Within a row superscripts without a common letter differ at P < 0.05. Significant P-values and trends are presented in bold*.

**Table 5:** Behavioral activities (% of total observations) at week 2 after weaning (42 days of age).

**Behavior at week 2 after weaning**	**DD**		**MO**		**Significance**		
	**CON**	**SUB**	**CON**	**SUB**	**DV**	**FP**	**DV × FP**
**“Ingestive behavior”**							
Eating feed	11.1 ± 0.6	11.3 ± 0.5	10.5 ± 0.6	11.7 ± 0.5	0.88	0.23	0.41
Drinking	2.0 ± 0.3	1.5 ± 0.3	1.9 ± 0.3	1.9 ± 0.3	0.49	0.40	**0.07^1^**
**“Exploratory behavior”**							
Exploring feed(er) and drinker	3.1 ± 0.3	3.3 ± 0.5	3.4 ± 0.5	3.8 ± 0.4	0.43	0.39	0.68
Exploring environment	18.8 ± 1.2^a^	29.4 ± 2.6^b^	28.3 ± 3.2^b^	26.5 ± 1.6^b^	**0.09**	0.04	**<0.01**
Nosing environment	9.5 ± 1.2	12.0 ± 1.1	11.7 ± 1.1	11.3 ± 0.7	0.47	0.22	0.18
Rooting environment	1.1 ± 0.2	2.0 ± 0.5	1.7 ± 0.5	2.2 ± 0.4	**0.04**	**0.03**	0.18
Chewing environment	6.6 ± 1.3^a^	13.1 ± 2.1^b^	12.8 ± 2.0^b^	10.5 ± 1.4^ab^	0.15	0.14	**<0.01**
Chewing air	1.7 ± 0.2	2.3 ± 0.4	2.0 ± 0.5	2.5 ± 0.5	0.64	0.23	0.75
**“Postures and locomotion”**							
Inactive behavior	50.5 ± 2.2^a^	40.5 ± 2.5^b^	43.6 ± 2.5^b^	41.1 ± 2.0^b^	0.17	**<0.01**	0.08
Standing and walking	2.8 ± 0.2	3.8 ± 0.2	3.1 ± 0.3	3.6 ± 0.2	0.73	**<0.01**	0.27
**“Pig-directed behavior”**							
Nosing pen mates	4.1 ± 0.3^a^	3.3 ± 0.2^ab^	2.9 ± 0.3^b^	3.6 ± 0.3^ab^	**<0.10**	0.98	**0.03**
Ear biting	0.4 ± 0.1	0.3 ± 0.1	0.4 ± 0.1	0.6 ± 0.1	0.32	0.88	0.19
Tail biting	0.6 ± 0.1	0.8 ± 0.3	0.5 ± 0.1	0.6 ± 0.1	0.32	0.48	0.81
Belly nosing	0.2 ± 0.1	0.2 ± 0.1	0.3 ± 0.2	0.4 ± 0.4	0.93	0.73	0.89
Manipulating pen mates	1.1 ± 0.2	0.7 ± 0.1	0.7 ± 0.1	0.9 ± 0.2	0.90	0.64	**0.09**^**1**^
Mounting pen mates	0.5 ± 0.2	0.3 ± 0.1	0.4 ± 0.2	0.3 ± 0.1	0.99	0.28	0.94
Aggression	0.1 ± 0.05	0.2 ± 0.1	0.2 ± 0.1	0.2 ± 0.1	0.70	0.63	0.48
**“Other behavior”**							
Playing	3.2 ± 0.4	2.5 ± 0.4	2.3 ± 0.4	3.3 ± 0.3	0.80	0.61	**0.03**^**1**^
Comfort behavior	0.3 ± 0.1	0.5 ± 0.1	0.6 ± 0.1	0.5 ± 0.1	0.11	0.54	0.29
Eliminating	1.0 ± 0.1	1.2 ± 0.2	0.8 ± 0.1	0.9 ± 0.2	**0.05**	0.37	0.76

*Piglets were provided with one solid feed item as monotonous diet (MO) or four solid feed items as diverse diet (DD) before weaning and their pre-weaning diet was presented without substrate (CON) or with substrate (SUB) in one of two feeders. DV, dietary variety; FP, feed presentation. Data are means ± SEM based on pen averages. Within a row superscripts without a common letter differ at P < 0.05. Significant P-values and trends are presented in bold*.

1 *No significant differences were observed using post-hoc pairwise comparisons of least squares means*.

#### Exploratory Behavior

DD-piglets spent less time exploring the feed(er) and drinker than MO-piglets at week 1 post-weaning (DD: 2.4 ± 0.2 vs. MO: 3.2 ± 0.3%). DD-piglets also explored their environment less than MO-piglets in this period (DD: 19.8 ± 1.0 vs. MO: 24.7 ± 1.3%), which was reflected by lower levels of chewing the environment (DD: 6.3 ± 0.8 vs. MO: 10.5 ± 1.2%) and chewing air (DD: 1.7 ± 0.2 vs. MO: 2.6 ± 0.2%). At week 2 post-weaning, interactions between DV × FP were found on exploring the environment (*P* < 0.01) and chewing the environment (*P* < 0.01), showing that DD-CON piglets spent less time exploring their environment than the other three treatment groups (*P* ≤ 0.01 for all) and DD-CON piglets had lower levels of chewing their environment than DD-SUB and MO-CON piglets (*P* < 0.01 for both). DD-piglets showed less rooting of their environment compared to MO-piglets (DD: 1.5 ± 0.3 vs. MO: 2.0 ± 0.3%) and SUB-piglets showed more rooting of their environment compared to CON-piglets (SUB: 2.1 ± 0.3 vs. CON: 1.4 ± 0.2%) at week 2 post-weaning. No effects were found on nosing the environment.

#### Postures and Locomotion

DD-piglets tended to be inactive for a larger amount of time than MO-piglets at week 1 post-weaning (DD: 46.7 ± 2.0 vs. MO: 41.9 ± 2.0%). CON-piglets were inactive for a larger amount of time than SUB-piglets at week 1 post-weaning (CON: 46.3 ± 2.0 vs. SUB: 42.3 ± 2.1%). Also in week 2 post-weaning, but only in DD (DV × FP, *P* = 0.08), as DD-CON piglets spent more time inactive than the other three treatment groups (*P* ≤ 0.03 for all). CON-piglets also showed less standing and walking at week 2 post-weaning than SUB-piglets (CON: 2.9 ± 0.2 vs. SUB: 3.7 ± 0.2%).

#### Pig-Directed Behavior

Nosing pen mates was higher for DD-piglets than MO-piglets at week 1 post-weaning (DD: 4.2 ± 0.3 vs. MO: 3.1 ± 0.2%). SUB-piglets had higher levels of manipulating pen mates (SUB: 0.8 ± 0.1 vs. CON: 0.5 ± 0.1%) and aggression (SUB: 0.2 ± 0.04 vs. CON: 0.1 ± 0.03%) than CON-piglets at week 1 after weaning. At week 2, DD-piglets also had higher levels of nosing pen mates than MO-piglets, but only within CON (DV × FP, *P* = 0.03). A trend for a DV x FP interaction was found on manipulating pen mates at week 2 after weaning (*P* = 0.09), but no significant differences were observed using *post-hoc* pairwise comparisons of least squares means. Ear biting, tail biting, belly nosing and mounting pen mates were not affected by treatments.

#### Other Behavior

DV × FP interacted on comfort behavior at week 1 post-weaning (*P* = 0.02), showing lower levels of comfort behavior for MO-SUB piglets in comparison with MO-CON (*P* = 0.03) and DD-SUB piglets (*P* = 0.01). In addition, DV × FP interacted on playing at week 2 post-weaning (*P* = 0.03), but no significant differences were observed using *post-hoc* pairwise comparisons of least squares means. Lastly, DD-piglets tended to eliminate more than MO-piglets at week 2 post-weaning (DD: 1.1 ± 0.1 vs. MO: 0.8 ± 0.1%).

### Body Lesions in the First 2 Days After Weaning

Dietary diversity before weaning reduced the number of body lesions at 4 h after weaning (DD: 15.8 ± 1.9 vs. MO: 24.1 ± 3.3 lesions), while feed presentation in substrate during lactation, in contrast, increased the number of body lesions (SUB: 23.7 ± 3.0 vs. CON: 16.1 ± 2.4 lesions; Table 6). A DV x FP effect was found on the number of body lesions at 24 and 48 h after weaning, showing that MO-SUB piglets had more lesions on their body than the other three treatment groups at 24 h after weaning, but no significant pairwise differences were observed at 48 h after weaning.

**Table 6:** Body lesions at 4, 24, 48 h and 15 days post-weaning of piglets provided with one solid feed item as monotonous diet (MO) or four solid feed items as diverse diet (DD) before weaning and their pre-weaning diet presented without substrate (CON) or with substrate (SUB).

**Body lesions**	**DD**		**MO**		**Significance**		
	**CON**	**SUB**	**CON**	**SUB**	**DV**	**FP**	**DV x FP**
4 h	14.5 ± 2.5	17.2 ± 2.8	17.8 ± 4.1	30.3 ± 4.5	**0.06**	**0.04**	0.29
24 h	3.2 ± 1.1^a^	1.8 ± 0.7^a^	2.4 ± 0.6^a^	6.5 ± 1.1^b^	**0.06**	0.37	**<0.01**
48 h	0.8 ± 0.3	1.8 ± 0.5	1.7 ± 0.5	1.0 ± 0.2	0.97	0.63	**0.04^1^**
d 15	4.8 ± 1.0	6.3 ± 0.9	3.2 ± 0.6	5.0 ± 1.1	**0.03**	**0.02**	0.47

*DV, dietary variety; FP, feed presentation. Data are means ± SEM based on pen averages. Within a row superscripts without a common letter differ at P < 0.05. Significant P-values and trends are presented in bold*.

1 *No significant differences were observed using post-hoc pairwise comparisons of least squares means*.

### Body Lesions and Damage at 2 Weeks Post-weaning

**Figure 7:**
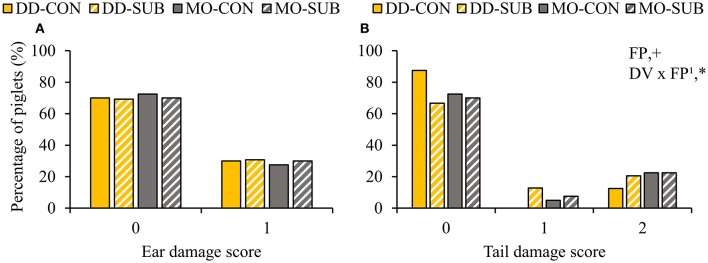
Occurrence of ear **(A)** and tail damage **(B)** (% of piglets with each score) at 2 weeks post-weaning on piglets provided with creep feed as a monotonous diet (MO) or four feed items simultaneously as a diverse diet (DD) before weaning and their pre-weaning diet was presented without (CON) or with substrate (SUB) in one of two feeders. DV, dietary variety; FP, feed presentation. Ear damage was classified as 0: no damage or 1: bite marks or small wound. Tail damage was classified as 0: no damage, 1: bite marks or 2: small or medium wound. Data are expressed as means. ^1^FP effect within DD. +, *Significant effects at *P* < 0.10 and <0.05, respectively.

Dietary diversity and feed presentation in substrate before weaning increased the number of body lesions at d15 after weaning (DD: 5.6 ± 0.7 vs. MO: 4.1 ± 0.6 lesions; SUB: 5.6 ± 0.7 vs. CON: 4.0 ± 0.6 lesions; Table 6). Dietary variety, feed presentation and their interaction did not affect the percentage of piglets with ear damage at 2 weeks post-weaning (Figure 7A). The percentage of piglets with tail damage, however, was affected by the interaction between DV × FP, as DD-CON piglets had less often higher tail damage scores than DD-SUB piglets (Figure 7B).

### Feed Intake After Weaning

Dietary variety and feed presentation tended to interact in their effect on feed intake in the first 4 h after weaning (Table 7), but no significant differences were found using *post-hoc* pairwise comparisons of least squares means. SUB-piglets had a lower feed intake on the second day post-weaning (SUB: 243 ± 18 vs. CON: 318 ± 13 g/d) and between d0–2 post-weaning (SUB: 186 ± 15 vs. CON: 241 ± 17 g/d, *P* = 0.02) vs. CON-piglets. They also had a lower ADFI between d0–5 (SUB: 286 ± 9 vs. CON: 324 ± 13 g/d, *P* = 0.03) and in total from d0–15 post-weaning (SUB: 500 ± 10 vs. CON: 537 ± 19 g/d). Dietary variety and feed presentation interacted in their effect on ADFI between d5–15 (*P* = 0.04). *Post-hoc* pairwise comparisons showed that DD-CON piglets had a higher ADFI in this period compared to the other three treatment groups (*P* ≤ 0.02 for all comparisons).

**Table 7:** Weaner performance in the first 2 weeks after weaning (d0–15 post-weaning).

	**DD**		**MO**		**Significance**		
	**CON**	**SUB**	**CON**	**SUB**	**DV**	**FP**	**DV × FP**
**FI, g/piglet**							
0–4 h	27 ± 2	11 ± 6	6 ± 2	13 ± 4	0.27	0.92	**<0.10^1^**
4–24 h	146 ± 35	121 ± 19	150 ± 25	112 ± 32	0.99	0.23	0.73
**ADFI, g/piglet/d**							
d0–1	172 ± 42	133 ± 23	156 ± 26	124 ± 34	0.76	0.21	0.99
d1–2	326 ± 23	264 ± 19	310 ± 15	222 ± 29	0.20	**<0.01**	0.58
d2–5	387 ± 20	357 ± 11	371 ± 21	347 ± 19	0.27	0.11	0.66
d5–15	668 ± 26^a^	605 ± 14^b^	618 ± 36^b^	610 ± 26^b^	**0.09**	**0.08**	**0.04**
Total, d0–15	556 ± 23	500 ± 11	517 ± 29	499 ± 18	0.10	**0.03**	0.11
**ADG, g/piglet/d**							
d0–1	−2 ± 95	−62 ± 54	83 ± 63	−139 ± 78	0.91	**0.04**	0.22
d1–2	474 ± 48	358 ± 29	467 ± 47	412 ± 36	0.62	**0.04**	0.34
d2–5	292 ± 29	301 ± 21	275 ± 19	318 ± 28	0.76	**0.07**	0.19
d5–15	508 ± 23^a^	453 ± 12^b^	474 ± 33^ab^	469 ± 27^ab^	0.98	0.19	**0.04**
Total, d0–15	429 ± 23^a^	382 ± 9^b^	407 ± 28^ab^	395 ± 20^ab^	0.91	**<0.10**	**0.09**
BW at d15, kg	13.79 ± 0.33^a^	13.09 ± 0.15^b^	13.19 ± 0.53^ab^	12.87 ± 0.33^ab^	0.91	**<0.10**	**0.09**
Feed conversion ratio, d0–15	1.31 ± 0.02	1.31 ± 0.02	1.28 ± 0.02	1.27 ± 0.02	**0.08**	0.94	0.43
**Fecal consistency and diarrhea**							
Fecal consistency score	0.41 ± 0.10	0.34 ± 0.06	0.35 ± 0.11	0.37 ± 0.10	0.94	0.44	0.70
# days with diarrhea	4.50 ± 0.89	3.90 ± 0.59	4.10 ± 1.22	4.10 ± 0.90	0.89	0.51	0.89
# days with watery diarrhea	1.70 ± 0.67	1.20 ± 0.47	1.20 ± 0.47	1.40 ± 0.65	0.96	0.56	0.59
% pens with watery diarrhea	50	70	50	50	0.62	0.62	0.39

*Piglets were provided with one solid feed item as monotonous diet (MO) or four solid feed items as diverse diet (DD) before weaning and their pre-weaning diet was presented without substrate (CON) or with substrate (SUB). DV, dietary variety; FP, feed presentation. Data are means ± SEM based on pen averages. Within a row superscripts without a common letter differ at P < 0.05. Significant P-values and trends are presented in bold*.

1 *No significant differences were observed using post-hoc pairwise comparisons of least squares means*.

### Piglet Growth and Fecal Consistency After Weaning

SUB-piglets lost weight on the first day after weaning compared to CON-piglets (SUB: −100 ± 47 vs. CON: 40 ± 56 g/d) and gained less on the second day after weaning (SUB: 385 ± 23 vs. CON: 470 ± 33 g/d; Table 7). They also had a lower ADG between d0–2 post-weaning (SUB: 142 ± 24 vs. CON: 255 ± 24 g/d, *P* < 0.01), but tended to have a higher ADG between d2–5 post-weaning (SUB: 309 ± 17 vs. CON: 283 ± 17 g/d). Taken together, no differences were found in ADG between d0–5 post-weaning (data not shown). Dietary variety and feed presentation interacted in their effect on ADG between d5–15 (*P* = 0.04) and d0–15 (*P* = 0.09) and BW at d15 post-weaning (*P* = 0.09). *Post-hoc* pairwise comparisons showed that SUB-piglets had a lower ADG compared to CON-piglets in these two periods and a lower BW at d15 post-weaning, but only when fed a diverse diet before weaning (*P* = 0.02 for all). DD-piglets tended to have a higher feed conversion ratio than MO-piglets after weaning (1.31 ± 0.01 vs. 1.28 ± 0.01). Dietary variety, feed presentation and their interaction did not affect the prevalence, duration and severity of (watery) diarrhea (Table 7).

## Discussion

We investigated the effects of dietary variety (vs. monotony, DD vs. MO) and feed presentation (hidden in sand as substrate or not, SUB vs. CON) before weaning on the feeding behavior and performance of piglets up to two weeks after weaning. Dietary diversity highly stimulated the feeding behavior of suckling piglets and, in contrast with piglets on a monotonous diet, all piglets were observed to eat feed prior to weaning. Presenting a part of the feed in substrate hardly increased foraging behavior before weaning, although piglets spent more time exploring and eating from the feeder with sand than from the feeder without sand. Dietary diversity (that was only given pre-weaning) only affected piglet development after weaning to a limited extent, but piglets that were given a diverse diet presented in feeders without substrate seemed to perform the best in the post-weaning period of the four groups in terms of feed intake, body weight gain and tail damage. Presenting the pre-weaning diet in substrate negatively affected weaner piglet performance, as post-weaning feed intake and growth were reduced and weaning-stress-induced behaviors were increased.

### Effects of Dietary Variety

Dietary diversity stimulated time spent exploring the feed and eating it by at least 2.5 times at all observation days. In total DD-piglets spent 14% of their time interacting with the feed of which they spent 12% of the time eating it at 4 weeks of age, and consumed 1267 g feed during lactation (determined in fresh weight), which seems exceptionally high compared to MO-piglets (5% time spent eating, 260 g feed intake) and previous studies (2% and 50–90 g/piglet in week 4 in Appleby et al. (50); 3.5–4.5% at week 3 and 601–693 g/piglet during lactation in van den Brand (51); 4.4% at week 4 and 397 g/piglet during lactation in Middelkoop et al., under review). Moreover, dietary diversity enhanced the percentage of piglets observed to be eating by 38% early in lactation and by 10% shortly before weaning and all DD-piglets were seen eating feed during lactation as compared with a monotonous diet. Dietary diversity particularly stimulated piglets to start eating early, as more than 75% of the piglets were observed eating from 11 days of age, whereas in MO it was 23%. The success of dietary variety on the feeding behavior of suckling piglets may be the result of a reduction in sensory-specific satiety that is induced by exposure to a monotonous diet. This is supported by our observation that 99% of the DD-piglets consumed more than one of the four solid feed items before weaning, of which 86% consumed all four feed items, thereby indicating that piglets prefer to eat diverse feed items when provided the choice. We observed a similar effect when piglets were given the choice between two feeds, in which 88% of the eaters consumed both (47). As a result, DD-piglets experienced variety in all senses, including sight (e.g., color, size, and shape), smell, taste (e.g., sweet, bitter), touch (e.g., texture), and hearing (e.g., crunch). The variety in feed items may have reduced sensory-specific satiety, resulting in an increase in feed intake. It should be noted that there were large individual differences in preferences for the different feed items, and in the strength of the preferences, as some piglets distributed their eating time more or less equally over the items, whereas others fed on one, two or three feed items mainly. The stimulation of feed intake could therefore also partly be the result of more choice allowing piglets to select their preferred item. The feeding behavior of DD-piglets may also have been enhanced by intrinsic exploration toward the feed (47, 51), elicited by differential sensorial experiences of the feeds. Our previous study in piglets suggests that feeds with multiple sensory differences can enhance feed exploration and intake more than feeds that vary in flavor only (47), whereas specific properties of the feed may also stimulate feed intake, such as a larger size (51). This mechanism is further supported by the positive correlation between time spent exploring the feed and eating it. Alternatively, post-ingestive signals may have mediated the increased feed intake by DD-piglets, as differences in nutrient profiles existed between the diets of DD- and MO-piglets. Changes in post-ingestive signals may lead to physiological changes in the animal, such as modification of appetite-controlling hormones and, as such, affected feed intake. Alternatively, the increase in feed intake caused by dietary diversity may have exerted changes in appetite-controlling hormonal profiles as result of changes in the feeding pattern of the animals, as discussed by Villalba et al. (44).

DD-piglets grew faster than MO-piglets in the last 2 days before weaning and thereby seems in agreement with lambs in a diversity treatment that tended to grow faster than lambs in the other monotonous treatments (44). Although DD-piglets spent four times more time on feeding on solids than mothers' milk, they continued to suckle milk and no differences were found in time spent suckling between DD- and MO-piglets. It is, therefore, suggested that the higher weight gain of DD-piglets compared to MO-piglets in the last 2 days prior to weaning might be the result of an earlier uptake of feed (thereby stimulating the development of the gastro-intestinal tract and gut microbiota) or a greater uptake of feed (resulting in a higher energy intake and/or heavier digesta in the gut). Both may play a role as bowls of DD-pens were refilled more often already from the first feeding phase onwards, and the metabolizable energy intake from solid feed by DD-piglets was roughly 3.4 times higher than the metabolizable energy intake from solid feed by MO-piglets. This difference in ME intake is sufficiently high to account for the higher growth in DD-piglets as compared with MO-piglets toward weaning. Using the method described by Pluske et al. (52), the contribution of solid feed to the total energy intake before weaning was estimated to be 11.9% for DD-piglets and 3.7% for MO-piglets. The energy intake from solid feed by DD-piglets could be explained for ~50, 28, 20, and 2% by the intake of peanuts, cereal honey loops, creep feed and celery, respectively. Time spent eating did not correlate with average daily gain between d26 and 28 in DD-piglets, while it did in MO-piglets. An underlying reason may be that the consumption of feed items, and thus nutrient and energy intake, was more variable between DD-piglets than between MO-piglets, of which the latter could only consume creep feed.

When given the choice between creep feed, celery, cereal honey loops and peanuts, piglets were seen more often eating peanuts at d18 and d27 than the other items, although peanuts were not refilled more often than celery and cereal honey loops. We therefore assume that the time piglets spent eating peanuts was longer, but not resulting in more actual intake than the other items, because piglets may have spent more time chewing on the peanuts to break the peanut into smaller pieces before ingestion could occur or to crack the shell before they could ingest the nuts. This may also have affected the correlation between time spent eating and average daily gain between d26 and 28. In addition, creep feed was less preferred to the other three feed items from d18 (based on behavioral observations and refills), which were larger in size and more complex in texture than the pellets. Larger feed items have been found previously to stimulate feed intake, as they are easier to handle for piglets (51), which may at least partly explain why creep feed was least preferred. It should be noted, though, that the four feed items differed in various sensory properties which may, apart from size differences, have affected preferences. Pen differences were also observed in these patterns, which may indicate social transmission of feed preferences within a pen, as piglets have been shown to acquire information concerning feed from their siblings (53), although a genetic influence on feed preferences cannot be excluded.

On top of a positive effect of dietary diversity on piglet performance before weaning, dietary diversity may also improve animal welfare before weaning, by providing individuals with food choices [as suggested in laying hens by Edgar et al. (54)] and stimulating playing with the feed. Play has been proposed to both induce and reflect positive welfare [reviewed by (55)], although this does not seem straightforward in all cases (56). The main potential advantage of a high feed intake before weaning is, however, that it may facilitate coping with weaning due to its expected benefits for post-weaning feed intake, body weight gain (24, 25), net absorption in the small intestine (57), gut physiology and gut microbiota development. This is of importance because several studies have shown that eaters outperform non-eaters (18, 58, 59) and good/early eaters outperform bad eaters (16, 60) in terms of feed intake and weight gain. Based on these studies, one would expect large beneficial effects of dietary variety on post-weaning feed intake and gain, as this treatment successfully stimulated solid feed intake and piglets to become eaters before weaning. Contrary to expectations, however, the beneficial effects were minor, as piglets that were given the diverse diet in two feeders without substrate during lactation only had the highest feed intake and body weight gain in the period from day 5 to 15 post-weaning. In terms of behavior, it could not be concluded whether the effects of dietary diversity on the behavioral development of piglets after weaning were beneficial for piglet welfare or not. Firstly, DD-piglets had a lower number of body lesions at 4 h after weaning compared to MO-piglets, which could point to a lower level of aggression. Aggression between unfamiliar piglets immediately after weaning is mainly aimed at establishing a new social hierarchy (61), but mixing of piglets was performed equally over treatments in this study. As frustration can also induce aggression in pigs (62), the lower number of body lesions at 4 h after weaning in DD piglets may potentially also reflect less frustration-related aggression as compared with MO-piglets. However, DD-piglets had a higher number body lesions than MO-piglets at 15 days after weaning. Secondly, DD-piglets spent less time exploring the feed(er) and drinker, chewing the environment (i.e., chain and parts of the pen), and chewing air (i.e., sham or vacuum chewing), but showed more nosing pen mates and tended to be inactive for a longer period of time at week 1 post-weaning compared to MO-piglets. Chewing on a chain (63), pen fixtures and/or air (64, 65), nosing pen mates (1, 66), and inactive behavior [e.g., (1, 64, 65)] have been found to increase in barren as compared with enriched housing, which is commonly thought to result from unfulfilled needs for exploration in the absence of suitable rooting substrates and may reflect stress. At week 2 post-weaning, effects seemed more pronounced in piglets that were fed a diverse diet in feeders without substrate, as they had the lowest level of chewing environment, highest level of nosing pen mates and being inactive and lowest number of piglets with higher tail damage scores.

One of the reasons that the beneficial effects of dietary diversity on post-weaning performance might have been minor, in spite of its impact on pre-weaning feed intake and the number of eaters, is that DD-piglets seemed to appreciate the creep feed the least compared to the other three feed items. They spent less time eating creep feed than MO-piglets at d18 and d27 and a lower number of DD-piglets were classed as creep feed eaters from d19. DD-piglets thereby mainly ingested the other feed items, which connected less well to the commercial weaner diet in terms of structure and ingredient composition. The latter may also explain the trend for a higher feed conversion ratio in DD-piglets that was found after weaning. The interaction between the composition of the pre- and post-weaning diet is one of the determinants of post-weaning performance (67) and may therefore play an important role in the success of a high feed intake before weaning on post-weaning performance. Secondly, it has been well-documented in children that they begin to show ‘picky eating' behavior, such as strong food preferences, when they are exposed to an increasingly diverse diet during weaning, as reviewed by Samuel et al. (68). Indeed, some DD-piglets were observed to develop strong feed preferences during exposure to the diverse pre-weaning diet, which could make them less willing to try the monotonous post-weaning diet than MO-piglets, resulting in a lower post-weaning feed intake than expected based on the high pre-weaning feed intake level that we observed. A third explanation for the limited post-weaning effects of DV may be the loss of diversity, as the diverse feed items were only provided before weaning, particularly because DD-piglets not only explored and ate the feed items, but also used them to play with. The loss of environmental enrichment has shown detrimental effects on pig welfare and production, also in early life (1, 69–71). Therefore, it is recommended to investigate the effects of dietary diversity by continuing the provision of the diverse diet after weaning or even strengthening the diversity in the diet after weaning, thereby creating a more gradual dietary change. The potential negative effects of loss of diversity may be less when a pre-weaning diet is provided that is less diverse, such as the two pellet types that were given as diverse diet in our previous study, but piglets were only followed up to weaning (47). We suggest that dietary diversity provided from the post-weaning period onwards may also be beneficial for piglet performance, but this warrants further investigation, as it has only been studied in 42-day old nursery piglets by performing flavor variety trials of 90 min (46).

### Effects of Feed Presentation

The provision of substrate, such as earth, wood bark, and the combination of wood shavings, straw, peat, and branches has been found to highly stimulate exploratory behavior (3, 70, 72). We therefore expected that supplementing (a part of) the pre-weaning diet with substrate may encourage litters to spend more time at the feeder to explore. SUB-piglets, however, only tended to spend more time on feed exploration than CON-piglets at d11, which may be because other studies used a larger amount of substrate and different substrate sources, which were edible, which is not the case for sand that was used as substrate in our experiment. We also expected that SUB would increase pre-weaning feed intake, but our data on the number of refills, time spent eating and eater classification do not support this, except that SUB-litters consisted of more good/early eaters than CON-litters when fed with creep feed, when classified based on home pen observations. This is a mild indicator that SUB has the potential to improve the early intake of piglets. These results correspond to the findings of Wood-Gush and Beilharz (72), in which early weaned piglets that had access to a trough with earth did not seem to have a higher intake of the feed provided, but the number of piglets in this group that was seen eating during observations was larger.

Presenting the feed in substrate before weaning did not positively, but even negatively affected piglet performance and behavior after weaning. This is shown by a reduced feed intake in the 2 weeks after weaning, a reduced body weight gain particularly in the first 2 days after weaning and a trend for a lower body weight at 2 weeks post-weaning. In terms of post-weaning behavior, SUB resulted in an increase in manipulation and aggression between pen mates at week 1 after weaning and a higher number of body lesions at 4 h and week 2 post-weaning. These results indicate that SUB-piglets had a higher level of frustration and a poorer adaptation to the post-weaning environment than CON-piglets, which also here likely resulted from losing enrichment as substrate was not provided any longer. Similar detrimental effects of losing enrichment were found previously [straw: (73); wood shavings plus straw: (69)], although one study reported that the provision of straw as pre-weaning enrichment tended to increase feed intake in the first 2 days after weaning and reduced body weight loss in this period compared to pre-weaning barren housing, although no straw was provided any longer after weaning. However, in terms of behavior, losing the straw at weaning reduced play and increased belly nosing (71). Moreover, no detrimental effects of losing a box with wood bark as enrichment from the pre- to post-weaning period were seen on body weight gain and skin lesions and even positive effects on salivary cortisol the first day after weaning were described (3). The detrimental effects of removing enrichment might reflect altered behavioral needs of pigs due to their prior rearing experience, as suggested by Day et al. (73). Continuing the feed presentation strategy in a foraging-stimulating context after weaning therefore deserves further attention.

Despite that SUB-piglets did not significantly spend more time on feed(er) exploration than CON-piglets, SUB-piglets did spent more time on exploration at the feeder with sand compared to the feeder without sand at all observation days. This may derive from intrinsic foraging needs and indicates that piglets are motivated to forage from early in lactation onwards. In addition, piglets spent more time eating at the feeder with sand than at the feeder without sand (except for the first observation day), which was more pronounced within MO. This may indicate that piglets are willing to “work” for feed by rooting through the substrate, while the same feed was freely available nearby at the same time. This is often referred to as “contrafreeloading,” and has been previously observed in older pigs (36, 37). However, to prove this phenomenon in suckling piglets, a feeder with substrate only would be needed as a control (next to the feeder with feed and feeder with feed plus sand). Moreover, Wood-Gush and Beilharz (72) reported that piglets mostly went to eat and drink after using an earth trough to forage, suggesting foraging is indeed an appetitive component for eating. After rooting the sand in our study, piglets may therefore have stayed at the feeder to eat, which may partly explain why piglets spent more time eating at the feeder with sand at d18 and d27 compared to the feeder without sand.

In conclusion, a diverse feeding regime for suckling piglets highly stimulated feed exploration, eating, feed intake, and the percentage of eaters from an early age onwards, and enhanced their growth toward weaning. Dietary diversity thus has the potential to get all suckling piglets to eat and to improve piglet performance and, potentially, their welfare before weaning. Feed presentation in a foraging-stimulating context, i.e., in substrate, only subtly stimulated exploratory behavior and the percentage of good eaters before weaning, but piglets seemed motivated to forage as they spent more time at the feeder with sand than the feeder without sand to explore and, to a lesser extent, eat. Against expectations, post-weaning benefits as result of pre-weaning dietary diversity were minor and detrimental effects of feed presentation in a foraging-stimulating context were found on post-weaning adaptation of piglets. This could be due to the loss of diversity and substrate piglets experienced at weaning, therefore the reinforcement of dietary diversity and feed presentation in substrate after weaning deserves further attention. Piglets that were provided dietary diversity in feeders without sand before weaning seemed to perform the best in the post-weaning period of the four groups. Piglets in this treatment did not experience loss of sand, but were positively affected by dietary diversity before weaning, despite the loss of diversity at weaning that may have partly suppressed the positive effects of dietary diversity.

## Data Availability Statement

The datasets generated for this study are available on request to the corresponding author.

## Ethics Statement

The animal study was reviewed and approved by the Animal Care and Use committee of Wageningen University and Research (Wageningen, Netherlands).

## Author Contributions

AM, BK, and JB designed the experiment. AM, MM, and JB conducted the experiment. AM analyzed the data, wrote the manuscript, and prepared the figures. JB advised on data analyses. MM, BK, and JB substantively revised the manuscript.

### Conflict of Interest

The authors declare that the research was conducted in the absence of any commercial or financial relationships that could be construed as a potential conflict of interest.
