# Supplementary material for: Pigs Like It Varied; Feeding Behavior and Pre- and Post-weaning Performance of Piglets Exposed to Dietary Diversity and Feed Hidden in Substrate During Lactation
Source: Front Vet Sci. 2019 Nov 19;6:408. doi: 10.3389/fvets.2019.00408 (PMC6877737; doi:10.3389/fvets.2019.00408)
Supplement: Supplementary file 1 [file Table_1.DOCX]

Supplementary Material

**Supplementary Table S1.** Nutrient profile of celery, cereal honey loops and peanuts in shell

| **Nutritional value per 100 g^1^** | **Celery** | **Cereal honey loops** | **Peanuts** | **Unit** |
| --- | --- | --- | --- | --- |
| Metabolisable energy | 60 | 1597 | 2614 | kJ |
| Water | 92 | 6 | 2.3 | g |
| Protein | 1 | 9 | 25.2 | g |
| Carbohydrates | 2 | 74 | 12.9 | g |
| of which mono/disaccharides | 1 | 29 | 4.8 | g |
| Polysaccharides | 1 | 45 | 8.1 | g |
| Fat | 0 | 3.5 | 51.7 | g |
| Fatty acids | 0 | 3.3 | 49.1 | g |
| of which saturated | 0 | 0.7 | 7.5 | g |
| Monounsaturated cis | 0 | 1.3 | 32.1 | g |
| Polyunsaturated | 0 | 1.4 | 9.5 | g |
| Fiber | 1.1 | 7 | 6.8 | g |
| Sodium | 60 | 550 | 0 | mg |
| Potassium | 320 | 270 | 757 | mg |
| Calcium | 80 | 456 | 56 | mg |
| Phosphorus | 40 | 320 | 456 | mg |
| Magnesium | 12 | 70 | 216 | mg |
| Iron | 0.5 | 8 | 1.9 | mg |
| Copper | 0.01 |  | 0.61 | mg |
| Selenium | 0 |  | 16 | μg |
| Zinc | 0.1 | 2 | 3.3 | mg |
| Iodine | 1.1 | 2.4 | 2.5 | μg |
| Retinol activity equivalents | 241 | 0 | 0 | μg |
| Retinol equivalents | 484 | 0 | 1 | μg |
| Beta-carotene | 2900 |  | 4 | μg |
| Lutein | 7200 |  | 5 | μg |
| β-cryptoxanthin |  |  | 1 | μg |
| Vitamin B1 | 0.08 | 0.91 | 0.16 | mg |
| Vitamin B2 | 0.15 | 1.2 | 0.08 | mg |
| Vitamin B6 | 0.1 | 1.2 | 0.158 | mg |
| Vitamin B12 | 0 | 2.1 | 0 | μg |
| Nicotinic acid | 0.8 | 14.9 | 17.5 | mg |
| Folate equivalents | 16 | 268.2 | 56.6 | μg |
| Folic acid | 0 | 146 | 0 | μg |
| Vitamin C | 25 | 0 | 0 | mg |
| Vitamin D | 0 | 4.2 | 0 | μg |
| Vitamin E | 0.2 |  | 6.4 | mg |
| Vitamin K | 29.3 |  |  | μg |

^1^as-fed, data from the Dutch Food Composition Database of the Dutch National Institute for Public Health and the Environment (2016)

**Supplementary Table S2.** Nutrient profile of the creep feed

| **Calculated nutrient composition^1^** | **Creep feed** |
| --- | --- |
| Net energy | 1180 |
| Dry matter | 891 |
| Starch | 290 |
| Sugars | 41 |
| Non-starch polysaccharides^2^ | 261 |
| Crude protein | 195 |
| Crude fat | 61 |
| Crude fiber | 44 |
| Crude ash | 57 |
| Calcium | 9.1 |
| Phosphorus | 6.1 |
| Sodium | 2.2 |
| Standardized ileal digestible lysine | 11.9 |
| Standardized ileal digestible methionine | 4.8 |
| Standardized ileal digestible threonine | 7.1 |
| Standardized ileal digestible tryptophan | 2.4 |

^1^According to CVB (2007). Nutrients are presented in g/kg dry matter, except for dry matter (g/kg) and net energy (kJ/100 g). Metabolisable energy is estimated to be 1595 kJ / 100 g (Noblet et al., 1995).

^2^Calculated as the difference between dry matter and the sum of starch, sugars, crude protein, crude fat and crude ash.

**References**

CVB. 2007. Veevoedertabel 2007: chemische samenstellingen en nutritionele waarden van voedermiddelen. Centraal Veevoederbureau, Den Haag, the Netherlands.

Dutch National Institute for Public Health and the Environment (RIVM). 2016. Dutch Food Composition Database (NEVO). NEVO online version 2016/5.0, RIVM, Bilthoven, the Netherlands. https://nevo-online.rivm.nl/

Noblet, J., Fortune, H., Shi, X.S. and Dubois, S. 1994. Prediction of net energy value of feeds for growing pigs. J. Anim. Sci. 72: 344-354.

**Supplementary Table S3.** Ingredient composition of the creep feed

| **Ingredient component^1^** | **%** |
| --- | --- |
| Wheat | 21.9 |
| Barley | 15 |
| Maize | 15 |
| Soy protein concentrate | 7 |
| Soybeans (heat treated) | 5 |
| Galacto-oligosaccharides | 5 |
| Potato protein | 4 |
| Sugarbeet pulp (dehydrated) | 4 |
| Oat hulls | 4 |
| Inulin | 4 |
| High-amylose starch (± 75% amylose) | 4 |
| Soybean oil | 3 |
| Blood meal (spray dried) | 2 |
| Dicalcium phosphate | 1.7 |
| Sucrose | 1.5 |
| Calcium carbonate | 1.0 |
| Sodium chloride | 0.5 |
| Premix^2^ | 0.5 |
| Potassium bicarbonate | 0.3 |
| L-lysine hydrochloride | 0.3 |
| DL-methionine | 0.2 |
| L-threonine | 0.04 |
| L-tryptophan | 0.04 |
| **Total** | **100** |

^1^Feed colorant Indigo carmine was included in the feed (5 g/kg feed).

^2^Vitamin and mineral premix (per kg of feed): vitamin A: 10000 IU, vitamin D3: 2000 IU, vitamin E: 40 mg, vitamin K: 1.5 mg, vitamin B1: 1 mg, vitamin B2: 4 mg, vitamin B6: 1.5 mg, vitamin B12: 0.02 mg, niacin: 30 mg, D-pantothenic acid: 15 mg, choline chloride: 150 mg, folate: 0.4 mg, biotin: 0.05 mg, iron: 100 mg, copper: 20 mg, manganese: 30 mg, zinc: 70 mg, iodine: 0.7 mg, selenium: 0.25 mg, anti-oxidant: 125 mg.

**Supplementary Table S4.** Behaviors of piglets after weaning

| Behavior | | Description |
| --- | --- | --- |
| ‘Ingestive behavior‘ | | |
|  | Eating feed | Eating or chewing feed (at the feeder) |
|  | Drinking | Drinking water from drinking trough |
| ‘Exploring feed(er) and drinker’ | | |
|  | Exploring feeder | Sniffing, touching (with snout), rooting or chewing on feeder |
|  | Exploring feed | Sniffing, touching (with snout) or rooting the feed in the feeder |
|  | Exploring drinking trough | Sniffing, touching (with snout) or chewing on drinking trough |
| ‘Exploring environment’ | |  |
|  | Nosing environment | Sniffing, touching (with snout) part of the pen (e.g. floor, wall) |
|  | Rooting environment | Rooting part of the pen, scraping floor with one the front legs |
|  | Chewing environment | Chewing or nibbling part of the pen, including chew object |
|  | Chewing air | Sham chewing (not chewing on part of the pen, feed or chew object) |
|  | Chewing feces | Chewing and/or swallowing feces |
| ‘Inactive behavior’ | | |
|  | Lying eyes closed | Lying on side or belly with eyes closed without performing any other described behavior |
|  | Lying eyes open | Lying on side or belly with eyes open, sitting or kneeling without performing any other described behavior |
| ‘Standing and walking’ | |  |
|  | Standing | Piglet is upright, standing, without performing any other described behavior |
|  | Walking | Piglet is walking, without performing any other described behavior |
| ‘Play behavior’ | | |
|  | Playing individually | Play activities that involve one player; running across pen, rolling, pivoting, tossing head, flopping, sliding, scampering, nudging |
|  | Playing socially | Play activities that involve more players; e.g. running, pivoting, scampering, sliding across pen together, play fighting. |
|  | Playing with chew object | (Energetically) shaking head with chew object in mouth |
| ‘Nosing pen mates’ | | |
|  | Nosing body | Sniffing, touching (with snout) part of the body of a pen mate excluding snout |
|  | Snout contact | Mutual snout contact with pen mate |
| ‘Pig-directed behavior’ | |  |
|  | Ear biting | Chewing, nibbling or sucking the ear of a pen mate (except ear tag) |
|  | Tail biting | Chewing, nibbling or sucking the tail of a pen mate |
|  | Belly nosing | Rubbing belly of a pen mate with ≥3 up and down movements of the snout or sucking the navel or skin of the abdominal area of a pen mate |
|  | Manipulating pen mates | Chewing, nibbling or sucking part of the body of a pen mate excluding ear, tail and abdominal area |
|  | Mounting pen mates | Standing on hind legs while having front legs on pen mate |
|  | Aggression | Aggressively ramming, pushing, head-knocking, lifting or biting a pen mate, including mutual fighting |
| ‘Other behavior’ | | |
|  | Comfort | Rubbing body against objects or pen mates, scratching body with hind legs or stretching (part of) body |
|  | Eliminating | Defecating or urinating |
